# Special Issue “Flavonoids and Their Disease Prevention and Treatment Potential”: Recent Advances and Future Perspectives

**DOI:** 10.3390/molecules25204746

**Published:** 2020-10-16

**Authors:** H.P. Vasantha Rupasinghe

**Affiliations:** 1 Department of Plant, Food, and Environmental Sciences, Faculty of Agriculture, Dalhousie University, Truro, NS B2N 5E3, Canada; vrupasinghe@dal.ca; Tel.: +1-902-893-6623; 2 Department of Pathology, Faculty of Medicine, Dalhousie University, Halifax, NS B3H 4R2, Canada

In recent years, the interest in flavonoids as dietary bioactives to prevent human diseases, as well as their candidacy as pharmaceutical leads, has exponentially expanded. Flavonoids are a sub-class of plant polyphenols that have been shown to possess numerous health-promoting physiological benefits in a wide range of investigations from cell-based assays to epidemiological and human intervention studies. In this e-book editorial, a brief overview and insights into articles published in the Special Issue of *Molecules* titled “Flavonoids and Their Disease Prevention and Treatment” are provided. It is evident that all these papers contribute toward new knowledge to discover and validate beneficial physiological functions and the therapeutic potential of dietary flavonoids. Beyond their role as biologically active molecules of plant food, new insights are presented for the potential use of flavonoid derivatives as effective therapeutics to manage certain cancers. Flavonoids also offer promising applications in the management of obesity- and inflammation-associated disorders as well as the control of infectious diseases, including COVID-19.

Flavonoids are ubiquitously present in plant-based foods and natural health products. The molecule of flavonoids is characterized by a 15-carbon skeleton of C6-C3-C6, with the different structural configuration of subclasses. The major subclasses of flavonoids with health-promotional properties are the flavanols or catechins (e.g., epigallocatechin 3-gallate from green tea), the flavones (e.g., apigenin from celery), the flavonols (e.g., quercetin glycosides from apples, berries, and onion), the flavanones (e.g., naringenin from citrus), the anthocyanins (e.g., cyanidin-3-*O*-glucoside from berries), and the isoflavones (e.g., genistein from soya beans). Scientific evidence has strongly shown that regular intake of dietary flavonoids in efficacious amounts reduces the risk of oxidative stress- and chronic inflammation-mediated pathogenesis of human diseases such as cardiovascular disease, certain cancers, and neurological disorders [1]. The physiological benefits of dietary flavonoids have been demonstrated to be due to multiple mechanisms of action, including regulating redox homeostasis, epigenetic regulations, activation of survival genes and signaling pathways, regulation of mitochondrial function and bioenergetics, and modulation of inflammation response. The role of flavonoids on gut microbiota and the impact of microbial metabolites of flavonoids on optimal health has begun to unravel. The complex physiological modulations of flavonoid molecules are due to their structural diversity. However, some flavonoids are not absorbed well, and their bioavailability could be enhanced through structural modifications and applications of nanotechnology, such as encapsulation. This Special Issue consists of four review articles on flavonoids and 13 original research articles, which cover the latest findings on the role of dietary flavonoids and their derivatives in disease prevention and treatment.

## 1. Dietary Flavonoids Exhibit Cancer Preventive Properties

Cancer chemoprevention is defined as the application of safe natural compounds, synthetic molecules, or their combinations to interfere in multistage carcinogenesis. In recent years, interest in plant-based food for reducing the risk of cancer has emerged as a realistic and cost-effective approach [2]. Some of the ancient plant food that has been used as nature’s medicine has now been investigated for their disease preventive and treatment properties. Haskap berry, also called blue honeysuckle (*Lonicera caerulea* L.), is a plant food used by Japanese native people for many curative purposes [3]. In this issue, we have made a milestone demonstration that dietary supplementation of haskap berry can suppress the carcinogen-induced lung tumorigenesis in A/JCr mice [4]. Supplementation of haskap berry either before or after the induction of tumors by intraperitoneal injection of tobacco-specific nitrosamine ketone reduced the lung tumor multiplicity, tumor area, and the expression of cancer proliferative biomarkers (proliferating cell nuclear antigen and Ki-67) in lung tissues. Haskap is a unique berry in that 80% of its anthocyanins are contributed by a single molecule, cyanidin-3-*O*-glucoside [5]. Hydroxybenzoic acids are the major degrative products as well as in vivo metabolites of cyanidin-3-*O*-glucoside [6]. The review written by Sankaranarayanan and colleagues discussed the role and mechanism of hydroxybenzoic acids in the inhibition of cancer cell proliferation [7]. The majority of dietary flavonoids are not absorbed in the intestinal lumen but are subject to degradation by colonic microbiome generating hydroxybenzoic acids such as 2,4,6-trihydroxybenzoic acid (2,4,6-THBA), 3,4-DHBA, 3,4,5-THBA, 4-HBA, 2,4-DHBA, 2,6-DHBA. Evidence suggests that these microbial flavonoid metabolites could contribute to the prevention of certain cancers [8]. Natural hydroxybenzoic acids are also found in many medicinal plants known to have anti-cancer properties. Cellular mechanisms of cancer prevention by flavonoid metabolites such as hydroxybenzoic acids are relatively underexplored, and further investigations are warranted to fully explore flavonoids and their microbial metabolites as anti-cancer molecules. 

## 2. Flavonoid Derivatives Exhibit Enhanced Proapoptotic Activity in Cancer Cells 

The dietary intervention of flavonoids, such as anthocyanins, as an anti-cancer therapy, has also been quite extensively studied using mouse models [9]. Tutino and colleagues have demonstrated that flavonoids extracted from grape skin possess anti-proliferative and proapoptotic activity in colon cancer cells in vitro [10] and the possible mechanism involved the changes in the membrane polyunsaturated fatty acid profile [11]. Regulation of the expression of 15-lipoxygenase-1 (15-LOX-1) and peroxisome proliferator-activated receptor gamma (PPAR-γ) by grape skin flavonoids has been observed; however, future investigations should aim at understanding the role of flavonoids in membrane lipid synthesis and degradation in relation to proapoptotic activity in cancer cells. Similarly, Ko and colleagues have shown that tangeretin, a citrus peel-derived flavonoid, inhibits breast cancer stem cell formation through the suppression of signal transducer and activator of transcription 3 (Stat3) signaling [12]. Cancer stem cells are responsible for chemoresistance and recurrence of many cancers. Therefore, targeting cancer stem cells by flavonoids has the potential to become a novel cancer therapy. A limitation of the therapeutic application of flavonoids is their poor absorption in intestinal lumen and uptake, rapid metabolism, and poor uptake by targeted tissues. Nevertheless, attention should also be paid to specific flavonoids that could be cancer-promoting through the activation of the Keap1/Nrf2/ARE pathway in cancer cells [13,14]. 

Recent investigations revealed that the structural modification of flavonoids such as acylation can improve their selective anti-proliferative activity against cancer cells [15] and anti-metastatic activity [16]. Chalcone is the precursor of flavonoids, and Pawlak and colleagues have shown that methoxy derivatives of 2′-hydroxychalcone exhibit anti-proliferative and proapoptotic activity on canine lymphoma and leukemia cells [17]. The two most active derivatives 2′-hydroxy-2″,5″-dimethoxychalcone and 2′-hydroxy-4′,6′-dimethoxychalcone had the greater proapoptotic potential due to the addition of two methoxy groups. The methoxy derivatives of chalcones triggered DNA damage in the cell lines (GL-1 B/T-cell leukemia) resistant to chalcone-induced apoptosis. The findings demonstrate the potential of flavonoids and their analogs to develop as anti-cancer therapeutics.

## 3. The Relevance of Flavonoids in Obesity Prevention

Obesity is characterized as an excessive accumulation of fat due to an imbalance of high energy intake and less energy expenditure. Dietary phytochemicals such as flavonoids have been shown to modulate lipid metabolism by increasing basal metabolic rate and thermogenesis [18]. Kim and colleagues have shown that flavonoids isolated from *Acer okamotoanum* inhibit adipocyte differentiation and promote lipolysis in the 3T3-L1 adipocytes [19]. The study also showed significant down-regulation of adipogenic transcription factors, such as γ-cytidine-cytidine-adenosine-adenosine-thymidine/enhancer-binding protein-α, -β, and PPAR-γ. In addition, the flavonoids significantly activated 5′-adenosine monophosphate-activated protein kinase (AMPK), leading to inhibition of triacylglyceride accumulation, potentially inactivating the key regulatory enzyme acetyl Co-A carboxylase (ACC). Flavonoids need to be further explored for their ability to regulate adipocyte differentiation, lipolysis, and AMPK signaling in relation to weight management. It is important to understand the efficacious dose, which may be dependent on various factors, including the individual’s body mass index, lifestyle, age, and gut microbiome composition and diversity, etc.

Obesity-associated high blood pressure or hypertension is a serious public health concern. Obesity is associated with an increased renin-angiotensin-aldosterone system (RAAS), which regulates blood pressure [20]. Certain dietary flavonoids possess the potential to inhibit angiotensin-converting enzyme (ACE) that is the key regulatory enzyme of the RAAS and therefore regulates the blood pressure [21]. Here, Kamkaew and colleagues have demonstrated vasodilatory effects and mechanisms of action of flavonoids present in a traditional medicinal plant, *Bacopa monnieri*, using intact endothelial rings of mesenteric arteries of rats [22]. Flavonoids (luteolin and apigenin) at 0.1–100 µM caused vasorelaxation in a concentration-dependent manner, potentially by inducing endothelial nitric oxide synthase (eNOS) phosphorylation at Ser1177, leading to nitric oxide (NO) production. It is interesting to note that flavonoids have about twice the potency of saponins as vasodilators. However, the application of dietary flavonoids in the management of high blood pressure needs to be assessed and validated using appropriate human clinical trials. 

The biological processes of aging and senescence are accelerated by obesity, linked to metabolic syndrome, and the risk of developing chronic diseases increases with age [23]. The extension of lifespan and healthspan is dependent on behavioral, pharmacologic, and dietary factors, which remain largely unknown [24]. However, non-nutrient dietary antioxidants such as flavonoids are considered potential anti-aging agents or lifespan essentials. The ability of flavonoids to modulate some of the evolutionarily conserved hallmarks of aging, including oxidative damage, inflammation, cell senescence, and autophagy, have been reported. Guo and colleagues have investigated the anti-aging potential of neohesperidin and its synergistic effects with other citrus flavonoids in extending the lifespan of *Saccharomyces cerevisiae* [25]. Authors postulated that autophagy promoted by the decreased target of rapamycin complex 1 (TORC1) signaling is critically important for a long chronological lifespan. This and many similar studies also suggest the important considerations to make for the best combination of flavonoids or what nature has given us in certain plant foods when designing new dietary supplements or selecting functional foods for regular consumption with the aim of optimal healthy aging. 

Obesity, an inflammatory disease, has reached epidemic levels worldwide and has become the modern socioeconomic burden of the 21st century. Affecting nearly 30% of the world population, obesity is a risk factor for many chronic diseases including type 2 diabetes mellitus, cardiovascular disorders, certain cancers, hypertension, non-alcoholic fatty liver disease, and steatohepatitis, consequentially impacting the quality of life [26]. Therefore, reducing obesity and associated life-threatening diseases has become an emerging area of health sciences. In this Special Issue, Sudhakaran and Doseff have contributed to a comprehensive review of the impact of dietary flavones on obesity-induced inflammation [27]. The common flavones are luteolin, apigenin, chrysin, baicalin, acacetin, orientin, and apigenin, which could be found in food sources such as citrus fruits, vegetables, herbs, and grains. Based on the preclinical studies, flavones exhibit a potential role in suppressing adipogenesis, inducing the browning of white adipocytes, modulating immune responses in the adipose tissues, and hindering obesity-induced inflammation, which has been positively correlated with an enhanced cancer incidence. This review also summarizes the crosstalk between adipocytes and macrophages. However, further investigations are required to further understand the molecular mechanisms responsible for the anti-obesogenic activity of flavones. 

Similarly, Ferraz and colleagues reviewed the therapeutic potential of flavonoids in pain and inflammation [28]. Pathological pain results from inflammation as well as peripheral nerve injury. In this review, the authors discussed the preclinical and clinical evidence on the analgesic and anti-inflammatory proprieties of flavonoids. Flavonoids can suppress the expression and activation of many inflammatory mediators such as interleukin (IL)-1β, tumor necrosis factor α (TNF-α), NO, cyclooxygenase-2 (COX-2), vascular endothelial growth factor (VEGF), and intercellular adhesion molecule-1 (ICAM-1). Interestingly, Zaragozá and colleagues showed the anti-inflammatory as well as immunomodulatory activities of dihydroflavones, flavones, and flavonols [29]. Based on the lipopolysaccharide (LPS)-stimulated whole-blood experimental model, the authors suggested that quercetin, naringenin, naringin, and diosmetin could have a potential therapeutic effect in the inflammatory process of cardiovascular disease. With this emerging intense scientific evidence for ameliorating inflammatory conditions by flavonoids and due to their safe and cost-effective attributes, novel flavonoid-inspired nutraceuticals and therapeutics have begun to enter into the medical shelves to improve human health.

## 4. Potential Anti-COVID-19, Anti-Infectious, Anti-Inflammatory, Immunity-Enhancing Properties of Flavonoids

The epidemic of the infection COVID-19 by an emerging coronavirus SARS-CoV-2 in December 2019 poses significant threats to global health security and the economy [30]. Currently, there is no registered treatment or effective COVID-19-related vaccine. Therefore, investigators across the world are exploring alternative methods to manage this novel coronavirus through prevention and control of the propagation and transmission of COVID-19, antiviral and anti-infectious treatments, natural and effective inhibitors of COVID-19 entry proteins such as ACE2, approaches to enhance host immune response against RNA viral infection, and passive immunotherapy among many others.

In this Special Issue, Ngwa and colleagues communicate the potential of flavonoid-inspired prophylactics or therapeutics against COVID-19 [31]. Based on in silico studies, the authors show that flavonoids such as caflanone, hesperetin, myricetin, and flavonoid derivatives such as Equivir can bind with high affinity to the spike protein, helicase, and protease sites on the ACE2 receptor. Interestingly, caflanone, a unique prenylated flavonoid of *Cannabis*, inhibits virus entry factors including tyrosine-protein kinase ABL-2, cathepsin L, cytokines (IL-1β, IL-6, IL-8, macrophage inflammatory protein 1α (Mip-1α), TNF-α), and lipid kinase PI4Kiiiβ, as well as tyrosine-protein kinase receptor AXL-2, which facilitates mother-to-fetus transmission of coronavirus. 

Uncontrolled microbial infection can lead to inflammation as a response to the activation of the innate immune system. The persistence of chronic inflammation can cause fatal diseases such as sepsis. The severe conditions of sepsis due to cytokine storm may result in multiple organ failure, including the lungs. The activation of toll-like receptors (TLR) by microbes or microbial peptides is the critical initial step in developing sepsis [32]. *Escherichia coli*-induced sepsis in rodents has been used as an effective experimental model to assess potential anti-inflammatory compounds. Using this approach, Chauhan and colleagues found that isorhamnetin has the potential to prevent Gram-negative sepsis. Isorhamnetin treatment has significantly enhanced survival and reduced proinflammatory cytokines in the serum and lung tissue of *E. coli*-infected mice. Using docking studies, the authors demonstrated that isorhamnetin can interact directly with the TLR4/myeloid differentiation factor 2 (MD-2) complex that recognizes LPS on Gram-negative bacteria; therefore, it has the potential to inhibit the TLR4 cascade, and thus systemic inflammation and cytokine storm-mediated organ injury.

Urinary tract infections (UTIs) are the second most common type of infection worldwide. The protective effects of flavonoids and phenolic acids present in cranberry (*Vaccinium macrocarpon*) against UTIs are reviewed in this Special Issue [33]. The review reveals that besides uropathogenic *E. coli*, other bacteria such as *Klebsiella pneumoniae* or the Gram-positive bacteria of Enterococcus and Staphylococcus genera seem to be widely involved in UTIs. The reported clinical trials provide substantial evidence of cranberry as total or partial therapeutic alternatives to antibiotics in UTIs. However, individual and/or case-dependent variations of effectiveness have been seen. A-type proanthocyanidins, the oligomeric and polymeric flavonoids, are reported to be responsible for these preventive effects against UTIs. Unabsorbed proanthocytanidins are metabolized by the colon microbiota to generate many low-molecular weight microbial metabolites that can be further absorbed [34]. Future studies need to be focused on understanding the antimicrobial activities of proanthocyanidin-microbial metabolites and metabotypes in relation to UTIs. 

In this Special Issue, Quintanilla-Licea and colleagues also discuss the activity of flavonoids isolated from *Lippia graveolens* Kunth (Mexican oregano) as an anti-protozoal agent against *Entamoeba histolytica*, which causes amebiasis, a serious public health problem in developing countries. The major flavonoids of the extracts were pinocembrin, sakuranetin, cirsimaritin, and naringenin, which showed IC_50_ ranging from 28 to 154 µg/mL. These findings provide the basis for the development of flavonoid-based therapeutics against infectious diseases.

## 5. Future Perspectives of Flavonoid Research

In conclusion, regardless of their broad and multi-potent pharmacological properties, flavonoids are present in low amounts in many dietary sources, most of them possess low water solubility, some of them are unstable under certain conditions, have low intestinal absorption and bioavailability, and possibly need high doses to show efficacy in human studies. Most of the reported in vitro investigations have used metabolically unrealistic high concentrations of flavonoids to demonstrate the targeted efficacy and mode of action; however, these findings need to be validated using standardized in vivo experimental models. Investigations are still lacking in demonstrating the therapeutic efficacy of standardized flavonoid products isolated from plant-sources using prospective human studies. Scale-up, consumer- and environment-friendly green technologies are required for producing cost-effective flavonoid-based natural health products. Flavonoid supplementation to cancer patients should be done cautiously since they could interfere with radiotherapy and various chemotherapies. Multidisciplinary research collaborations are required in investigating enhanced and safe delivery systems of flavonoids to overcome bioavailability limitations, targeted delivery, and improve the therapeutic efficacy of certain flavonoids. Phase 2 metabolism and pharmacokinetics of most of the major flavonoids have been reported. However, the interaction of unabsorbed flavonoids with colon microbiota and resulting metabolites and their role in disease prevention and treatment still need to be understood. Thus, flavonoid nanotechnology, flavonoid-microbiome pharmacology, and flavonoid-inspired therapeutics remain an emerging discipline in life science. We welcome such novel investigations to present through MDPI *Molecules* special issue **Flavonoids and Their Disease Prevention and Treatment—2021.**

## References

[1] Williamson G., Kay C.D., Crozier A. (2018). The Bioavailability, Transport, and Bioactivity of Dietary Flavonoids: A Review from a Historical Perspective. Compr. Rev. Food Sci. Food Saf..

[2] George V.C., Dellaire G., Rupasinghe H.P.V. (2017). Plant Flavonoids in Cancer Chemoprevention: Role in Genome Stability. J. Nutr. Biochem..

[3] Rupasinghe H.P.V., Arumuggam N., Amararathna M., De Silva A.B.K.H. (2018). The Potential Health Benefits of Haskap (*Lonicera caerulea* L.): Role of Cyanidin-3-*O*-glucoside. J. Funct. Foods.

[4] Amararathna M., Hoskin D.W., Rupasinghe H.P.V. (2020). Cyanidin-3-*O*-Glucoside-Rich Haskap Berry Administration Suppresses Carcinogen-Induced Lung Tumorigenesis in A/JCr Mice. Molecules.

[5] Amararathna M., Hoskin D.W., Rupasinghe H.P.V. (2020). Anthocyanin-rich Haskap (*Lonicera caerulea* L.) Berry Extracts Reduce Nitrosamine-induced DNA Damage in Human Normal Lung Epithelial Cells In Vitro. Food Chem. Toxicol..

[6] Pace E., Jiang Y., Clemens A., Crossman T., Rupasinghe H.P.V. (2018). Impact of Thermal Degradation of Cyanidin-3-*O*-Glucoside of Haskap Berry on Cytotoxicity of Hepatocellular Carcinoma HepG2 and Breast Cancer MDA-MB-231 Cells. Antioxidants.

[7] Sankaranarayanan R., Kumar D.R., Patel J., Bhat G.J. (2020). Do Aspirin and Flavonoids Prevent Cancer through a Common Mechanism Involving Hydroxybenzoic Acids?—The Metabolite Hypothesis. Molecules.

[8] Thilakarathna W.P.D.W., Rupasinghe H.P.V. (2019). Microbial Metabolites of Proanthocyanidins Reduce Chemical Carcinogen-induced DNA Damage in Human Lung Epithelial and Fetal Hepatic Cells In Vitro. Food Chem. Toxicol..

[9] Rupasinghe H.P.V., Arumuggam N. (2019). Chapter 5: Health Benefits of Anthocyanins. Anthocyanins from Natural Sources.

[10] Tutino V., Gigante I., Scavo M.P., Refolo M.G., Nunzio V.D., Milella R.A., Caruso M.G., Notarnicola M. (2020). Stearoyl-CoA Desaturase-1 Enzyme Inhibition by Grape Skin Extracts Affects Membrane Fluidity in Human Colon Cancer Cell Lines. Nutrients.

[11] Tutino V., Gigante I., Milella R.A., De Nunzio V., Flamini R., De Rosso M., Scavo M.P., Depalo N., Fanizza E., Caruso M.G. (2020). Flavonoid and Non-Flavonoid Compounds of Autumn Royal and Egnatia Grape Skin Extracts Affect Membrane PUFA’s Profile and Cell Morphology in Human Colon Cancer Cell Lines. Molecules.

[12] Ko Y.-C., Choi H.S., Liu R., Kim J.-H., Kim S.-L., Yun B.-S., Lee D.-S. (2020). Inhibitory Effects of Tangeretin, a Citrus Peel-Derived Flavonoid, on Breast Cancer Stem Cell Formation through Suppression of Stat3 Signaling. Molecules.

[13] Fernando W., Rupasinghe H.P.V., Hoskin D.W. (2019). Dietary Phytochemicals with Anti-oxidant and Pro-oxidant Activities: A Double-edged Sword in Relation to Adjuvant Chemotherapy and Radiotherapy?. Cancer Lett..

[14] Suraweera T.L., Rupasinghe H.P.V., Dellaire G., Xu Z. (2020). Regulation of Nrf2/ARE Pathway by Dietary Flavonoids: A Friend or Foe for Cancer Management?. Antioxidants.

[15] Fernando W., Coombs M.R.P., Hoskin D.W., Rupasinghe H.P.V. (2016). Docosahexaenoic Acid-acylated Phloridzin, a Novel Polyphenol Fatty Acid Ester Derivative, is Cytotoxic to Breast Cancer Cells. Carcinogenesis.

[16] Fernando W., Coyle K., Marcato P., Rupasinghe H.P.V., Hoskin D.W. (2019). Phloridzin Docosahexaenoate, a Novel Fatty Acid Ester of a Plant Polyphenol, Inhibits Mammary Carcinoma Cell Metastasis. Cancer Lett..

[17] Pawlak A., Henklewska M., Hernández Suárez B., Łużny M., Kozłowska E., Obmińska-Mrukowicz B., Janeczko T. (2020). Chalcone Methoxy Derivatives Exhibit Antiproliferative and Proapoptotic Activity on Canine Lymphoma and Leukemia Cells. Molecules.

[18] Rupasinghe H.P.V., Sekhon-Loodu S., Mantso T., Panayiotidis M.I. (2016). Phytochemicals in Regulating Fatty acid β-Oxidation: Potential Underlying Mechanisms and Their Involvement in Obesity and Weight Loss. Pharmacol. Ther..

[19] Kim J.H., Lee S., Cho E.J. (2020). Flavonoids from *Acer okamotoanum* Inhibit Adipocyte Differentiation and Promote Lipolysis in the 3T3-L1 Cells. Molecules.

[20] Tanaka M. (2020). Improving Obesity and Blood Pressure. Hypertens. Res..

[21] Balasuriya B.W.N., Rupasinghe H.P.V. (2011). Plant Flavonoids as Angiotensin Converting Enzyme Inhibitors in Regulation of Hypertension. Funct. Foods Health Dis..

[22] Kamkaew N., Paracha T.U., Ingkaninan K., Waranuch N., Chootip K. (2019). Vasodilatory Effects and Mechanisms of Action of *Bacopa monnieri* Active Compounds on Rat Mesenteric Arteries. Molecules.

[23] Bonomini F., Rodella L.F., Rezzani R. (2015). Metabolic Syndrome, Aging and Involvement of Oxidative Stress. Aging Dis..

[24] Russo G.L., Spagnuolo C., Russo M., Tedesco I., Moccia S., Cervellera C. (2020). Mechanisms of Aging and Potential Role of Selected Polyphenols in Extending Healthspan. Biochem. Pharmacol..

[25] Guo C., Zhang H., Guan X., Zhou Z. (2019). The Anti-Aging Potential of Neohesperidin and Its Synergistic Effects with Other Citrus Flavonoids in Extending Chronological Lifespan of *Saccharomyces Cerevisiae* BY4742. Molecules.

[26] Blüher M. (2019). Obesity: Global Epidemiology and Pathogenesis. Nat. Rev. Endocrinol..

[27] Sudhakaran M., Doseff A.I. (2020). The Targeted Impact of Flavones on Obesity-Induced Inflammation and the Potential Synergistic Role in Cancer and the Gut Microbiota. Molecules.

[28] Ferraz C.R., Carvalho T.T., Manchope M.F., Artero N.A., Rasquel-Oliveira F.S., Fattori V., Casagrande R., Verri W.A. (2020). Therapeutic Potential of Flavonoids in Pain and Inflammation: Mechanisms of Action, Pre-Clinical and Clinical Data, and Pharmaceutical Development. Molecules.

[29] Zaragozá C., Villaescusa L., Monserrat J., Zaragozá F., Álvarez-Mon M. (2020). Potential Therapeutic Anti-Inflammatory and Immunomodulatory Effects of Dihydroflavones, Flavones, and Flavonols. Molecules.

[30] Zhang L., Liu Y. (2020). Potential Interventions for Novel Coronavirus in China: A Systematic Review. J. Med. Virol..

[31] Ngwa W., Kumar R., Thompson D., Lyerly W., Moore R., Reid T.-E., Lowe H., Toyang N. (2020). Potential of Flavonoid-Inspired Phytomedicines against COVID-19. Molecules.

[32] Hotchkiss R.S., Moldawer L.L., Opal S.M., Reinhart K., Turnbull I.R., Vincent J.-L. (2016). Sepsis and Septic Shock. Nat. Rev. Dis. Primers.

[33] González de Llano D., Moreno-Arribas M.V., Bartolomé B. (2020). Cranberry Polyphenols and Prevention against Urinary Tract Infections: Relevant Considerations. Molecules.

[34] Rupasinghe H.P.V., Parmar I., Neir S.V. (2019). Biotransformation of Cranberry Proanthocyanidins to Probiotic Metabolites by *Lactobacillus rhamnosus* Enhances Their Anticancer Activity in HepG2 Cells In Vitro. Oxidative Med. Cell. Longev..

